# Food web structure of parasitoids in greenhouses is affected by surrounding landscape at different spatial scales

**DOI:** 10.1038/s41598-019-44857-1

**Published:** 2019-06-11

**Authors:** Zhaoke Dong, Xingyuan Men, Shuang Liu, Zhiyong Zhang

**Affiliations:** 1 Beijing Key Laboratory of New Technology in Agricultural Application, National Demonstration Center for Experimental Plant Production Education, Beijing University of Agriculture, Beijing, 102206 China; 2 Institute of Plant Protection, Shandong Academy of Agricultural Sciences, Ji’nan, 250100 China

**Keywords:** Food webs, Entomology

## Abstract

Landscape management affects species interactions and can have notable effects on food web structure. Local parasitoid populations in greenhouses usually migrate from outside crops; biological control of greenhouse aphids may be thus highly dependent on the composition of surrounding landscape. However, it is less clear how surrounding landscape composition affects primary-hyperparasitoid food webs and pest control services in greenhouses. We investigated the food web of parasitoids on melon-cotton aphid (*Aphis gossypii* Glover) in watermelon greenhouses in two suburban Beijing counties over two years. We used the quantitative food web metrics (generality, vulnerability, link density, and interaction evenness) to assess the effects of landscape composition on primary-hyperparasitoid food web structure. We found that landscape with more cropland within 1–3 km tended to have more primary parasitoids per hyperparasitoid species (generality). Higher proportions of woodland at the 0.5 km scale were negatively correlated with the mean numbers of hyperparasitoid per primary parasitoid species (vulnerability), as well as with hyperparasitism rate and hyperparasitoid richness. Link density, interaction evenness and aphid mortality caused by parasitoids (parasitism rate) were not affected by landscape factors. However, active primary parasitism (biocontrol potential) increased with the proportion of woodland. This suggested that the bottom-up effect induced by primary parasitoids might benefit hyperparasitoids, thus exerting little influence of primary parasitoids on pest control. The top-down effect of hyperparasitoids may reduce with increasing woodland proportion. To enhance the effects of primary parasitoids, landscape management programs should also target, and thus limit the impact of hyperparasitoids.

## Introduction

In landscape ecology and biological pest control, the effects of landscape composition on species interactions and ecological functions are important topics^1–3^. Empirical work demonstrates that changes in landscape structure can alter host-parasitoid dynamics, affecting populations at a large scale^4^. Food web, which depicts network of trophic relationships in ecological community, has become a major concern in many studies. The diversity and complexity of food webs are considered to be important factors determining ecosystem function and stability^5^. The quantitative food web metrics can give a robust description of community structure, providing insights into the dynamic processes that structure ecological communities^6^. In agricultural landscape, agricultural intensification and the loss of semi-natural habitats can reduce biodiversity and associated biological control services^7–9^. Empirical study on the food-webs of cereal aphids across a landscape complexity gradient has shown that aphid-parasitoid food webs are less complex, with higher rates of parasitism in complex landscapes^10^. Understanding of food web structures and their responses to environmental factors can have implication for population management of pest and natural enemy^11,12^.

In most studies with parasitoids of agricultural pests, parasitoid species richness and/or parasitism rates in the crop habitat increases with landscape complexity (^13–16^, but see^17,18^). The percentage of arable land (or non-crop habitat) in a landscape is commonly used to indicate the landscape complexity^15,19^. Natural enemies in crop fields often benefit from the presence of non-crop habitats in landscapes, as these provide key resources such as alternative hosts, food resources, shelter and/or overwintering habitats^20,21^. Hyperparasitoids should be considered as a new target in biological control^22^. They disrupt biological control of aphids mediated by primary parasitoids^23,24^, and also may take advantage of non-crop habitats in a complex landscape^15,22^. That makes the effects of landscape context on biological control efficiency uncertain^25,26^. It is suggested that community and interaction structures might not be detected in studies that focus simply on species richness and abundance^27^. Thus assessing the landscape effects on community level is necessary. Food web interactions have previously been studied for aphid–parasitoid systems in the open field landscape context (e.g.^10^), but aphid-parasitoid interactions in greenhouses have not been explicitly explored, especially with respect to hyperparasitoid ecology.

As greenhouse crops are grown under controlled condition, greenhouse ecosystems are often very simple with low biodiversity. Stable environmental conditions are apparently conducive to some pests and diseases^28^. The general experience is that infestation by several small pest species cannot be avoided^29^. Greenhouses have very diversified structures, including sheet glass, fiberglass or plastic. Compared to glasshouses which are rather closed units, plastic greenhouses are more openly structured creating a constant interchange of pests and beneficials between the greenhouse crops and the neighboring outdoor crops and weeds^30^. Plastic greenhouses are common in temperate zones, e.g. China. Polyphagous pests which can feed on many crop plants and wild plants quickly become prevalent in plastic greenhouses. A polyphagous aphid, *Aphis gossypii* Glover, damages cucurbits and hundreds of other crops^31^. Therefore, it can move from surrounding plants to the greenhouse crops. Previous study showed that *A*. *gossypii* could colonize several different hosts successively with seasonal change^32^. The immigration of aphids into the greenhouse usually causes sudden and largely unpredictable density increases. The parasitoids can also move from surroundings to greenhouses, so the parasitoids in greenhouses have a certain degree of species diversity^33^. Therefore, biological control programs should consider not only releasing natural enemies in the greenhouses, but also attempting to conserve local populations in the surrounding areas^28^.

Little is known about how surrounding landscape composition affects food webs of (hyper)parasitoids in greenhouses. To address this knowledge gap, we investigated the primary parasitoids and hyperparasitoids of melon-cotton aphid (*A*. *gossypii*) in watermelon greenhouses located on the suburban plain of Beijing, China. *A*. *gossypii* is the only aphid species infesting watermelon in the Beijing area^32,34^. In this area, the management of *A*. *gossypii* under greenhouses conditions is based on chemical control. Nowadays, several biological control programs are under development and these include the identification of potential natural enemies. We calculated the food web metrics of primary-hyperparasitoid communities to test the following hypotheses: (i) the migration of parasitoids from surrounding croplands into greenhouses increases the food web complexity; (ii) landscape composition affects the primary parasitoids and hyperparasitoids differently; (iii) aphid control efficiency is related to food web structure.

## Materials and Methods

### Study sites and sampling

Sampling was conducted in Daxing and Shunyi counties, suburban Beijing, northern China. These two counties, which are 70 km apart, are located on plains that consist primarily of cultivated fields. There were six different sampling sites in Daxing and five in Shunyi: each site was in a different village. In general, sites were >3.0 km apart; one pair of sites was 1.2 km apart. Sampling sites varied slightly between 2015 (four in Daxing and five in Shunyi) and 2016 (five in Daxing and four in Shunyi). At each site, we sampled three to five neighboring watermelon greenhouses in the center of the landscape. Plastic greenhouses prevailed in our study area. These relatively open structures allowed pests and natural enemies to move between greenhouse crops and field crops.

At each site, we collected mummified aphids from as many watermelon plants as possible. Sampling was conducted twice between May and June of 2015, and three times between May and June of 2016. During each sampling event, we collect leaves with aphids and mummified aphids for 1 h per site. The GPS coordinates of each site were recorded using a Magellan handheld GPS receiver, a latitude-longitude projection. Sampling locations were confirmed by consulting topographical maps of the study area and Google Earth.

After collection, watermelon leaves were transported to our laboratory. Leaves were placed in fine-mesh cages and maintained at 25 ± 1 °C, with 70% relative humidity and a 14:10 light:dark photoperiod. Leaves were checked daily for parasitoid emergence. All of the mummies were removed from leaves and kept in gelatine capsules for the later identification of emerging adult parasitoids. As the different genera of primary parasitoids can be distinguished by mummy characters^35^, it was possible to identify the relationships between primary and hyperparasitoids. Emerging parasitoids were promptly placed in 95% ethanol and stored at −20 °C. Specimens were identified to species by comparison with materials that had been previously authoritatively identified and with the aid of taxonomic keys^36–39^. Morphological identification of specimens was performed by the Laboratory of Zoological Systematics and Evolution (IZCAS: Institute of Zoology, Chinese Academy of Sciences, Beijing). We used DNA barcoding to confirm morphological identifications^33^. We used the parasitism rate to reflect parasitoid-associated aphid mortality rate which was calculated as proportion of all parasitoids from all aphids (including mummies). In addition, we used the active primary parasitism to reflect the biocontrol potential which was calculated as the ratio of primary parasitoids (nonparasite) to all aphids. Hyperparasitism rates were calculated as the proportion of hyperparasitoid mummies from all mummies (including primary and hyperparasitoids).

### Quantitative food web structure

We pooled the data by site in each year, and analyzed the quantitative structures of primary-hyperparasitoid food web for each site. We calculated four food web metrics namely generality, vulnerability, link density and interaction evenness (for detailed formulae see^12,40^. Generality is the weighted mean number of host species (primary parasitoid) per consumer species (hyperparasitoid). Vulnerability is the weighted mean number of consumers (hyperparasitoid) per host species (primary parasitoid). Link density is the weighted mean number of links per specie which represents the ratio of the number of trophic interactions to the number of species. Interaction evenness are analogous to Shannon evenness, but with trophic interaction instead of species as the base unit^41^. These metrics are often used as measures of food web complexity.

### Landscape analysis

Land cover maps of sampling sites were obtained from a digital map provided by the ZiYuan-3 survey satellite (ZY-3) with 5-m resolution. Landscape structure was analyzed using ArcGIS 10 (ESRI, Redlands, CA, USA). Land covers were classified into seven general categories, including: cropland, vegetable, orchard, grassland, woodland, water, and urban. Cropland was mainly in cultivation with crops such as winter wheat and soybean. Vegetable contained protected fields, e.g. greenhouses in which vegetable were usually planted. The orchard had perennial crops, such as orchard trees. The grassland was grass dominated land. Woodland included all forest and shrub landing excluding the trees in residential sites. Water represented the wetland and open water. Urban represented human architecture and roads etc. For each of the sampling sites, landscape composition was estimated in four circular sectors (0.5, 1, 2 and 3 km radii), representing a nested set of landscape sectors at four spatial scales. The percentage of each category was measured within each landscape circle. At the 0.5 km scale, percentage cropland was negatively correlated with percentage woodland and percentage vegetable, while at 1 km scale, percentage cropland was only negatively correlated with percentage vegetable. At 2 km and 3 km scales, percentage cropland was strongly positively correlated with percentage grassland (see supplementary material Table S2).

### Statistical analysis

All analyses were performed in the statistical program R version 3.4.3^42^. We firstly analyzed the influence of year and county on each food web metrics, richness of primary parasitoid, richness of hyperparasitoid and (hyper)parasitism rates by using one-way analysis of covariance (ANCOVA). Then we analyzed the landscape effects on food web metrics as well as richness of primary parasitoid, richness of hyperparasitoid and (hyper)parasitism rates using linear mixed-effect models with county and year as random effects (package “lme4”^43^). Arcsine-square-root transformations were used for parasitism data to meet assumptions of the approach. Explanatory landscape variables in the full models were percentages of cropland, vegetable, orchard, grassland and woodland. To avoid the multicolinearity, we selected the landscape variables with a restriction that models with one landscape variable did not include the other correlated landscape variables. The analyses were conducted at each spatial scale separately. We performed an automated model selection (dredge function) based on Akaike’s information criterion for small sample sizes (AICc), and ranked alternative models using model probabilities known as Akaike weights (wi) (package “MuMIn”^44^). If one of the models was strongly supported (i.e., wi > 0.90), we based inferences on that model. However, in cases where no single model was superior, coefficients for each predictor variable were derived from the competing models using a model averaging procedure^45^. To test for spatial autocorrelation, we computed Moran’s I test for the residuals of the best-fitting models for each response variable (package “spdep”^46,47^). The analysis revealed no spatial autocorrelation. We used the packages ‘bipartite’^48^ for plotting food web structures.

## Results

### Effect of year and county

In total, 3911 parasitoids were recorded across all of the sites, of which 84.7% were primary parasitoids, and 15.3% hyperparasitoids. The food web metrics: generality, vulnerability and interaction evenness varied noticeably between the two years, but were similar between counties. In contrast, link density was significantly affected by county but not by year (Table 1). Primary parasitoid richness and hyperparasitism rate varied significantly between years, while the aphid mortality, active primary parasitism and hyperparasitoid richness were not affected by year or county (Table 1).

**Table 1:** F-values and levels of significance from general linear models relating food web metrics (generality, vulnerability, link density and interaction evenness), aphid mortality, active primary parasitism, hyperparasitism and richness of (hyper)parasitoids to factors such as year and county. Significant values (*p* < 0.05) are highlighted in bold.

Variable	Year		County	
	F_1,15_	*p*	F_1,15_	*p*
Generality	11.28	**0**.**004**	0.48	0.500
Vulnerability	8.47	**0**.**011**	3.18	0.095
Link density	0.02	0.894	9.73	**0**.**007**
Interaction evenness	9.50	**0**.**008**	3.95	0.066
Parasitism rate	3.36	0.087	0.29	0.601
Active primary parasitism	1.24	0.283	0.74	0.405
Hyperparasitism	9.13	**0**.**009**	2.27	0.153
Richness of primary parasitoids	27.74	**<0**.**001**	0.80	0.385
Richness of hyperparasitoids	<0.001	1.000	2.77	0.117

**Figure 1:**
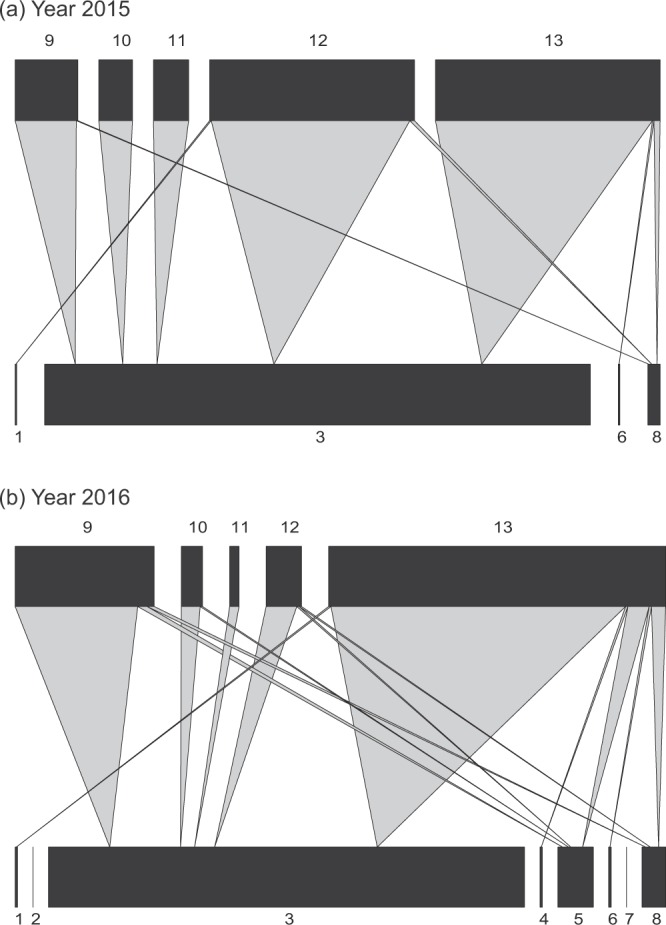
Quantitative host–parasitoid food webs for the year 2015 (**a**) and year 2016 (**b**). Lower bars represent host abundance (primary parasitoids); upper bars represent hyperparasitoid abundance (drawn at different scales). The width at the basis of the wedges reflects the pooled frequency of each host-parasitoid interaction. Species codes and abundances are given in Supplementary Table S1.

The quantified food webs of each year were pooled separately and are depicted in Fig. 1 (see supplementary material Table S1 for species names and abundances). The dominant primary parasitoid was *Binodoxys communis* (Gahan) (Fig. 1; code 3). The dominant hyperparasitoid species were *Pachyneuron aphidis* (Bouche) and *Syrphophagus aphidivorus* (Mayr) (Fig. 1; code 12 and code 13). The quantitative food webs (Fig. 1) showed that the remarkable changes occurred between years. Food webs in 2015 were dominated by two trophic interactions (two hyper parasitoids *P*. *aphidis* and *S*. *aphidivorus* parasitizing *B*. *communis*; code 12, 13 and 3 in Fig. 1). Food webs in 2016 were dominated by a single trophic interaction (a hyper parasitoid *S*. *aphidivorus* parasitizing *B*. *communis*; code 13 and 3 in Fig. 1).

### Landscape effects

**Figure 2:**
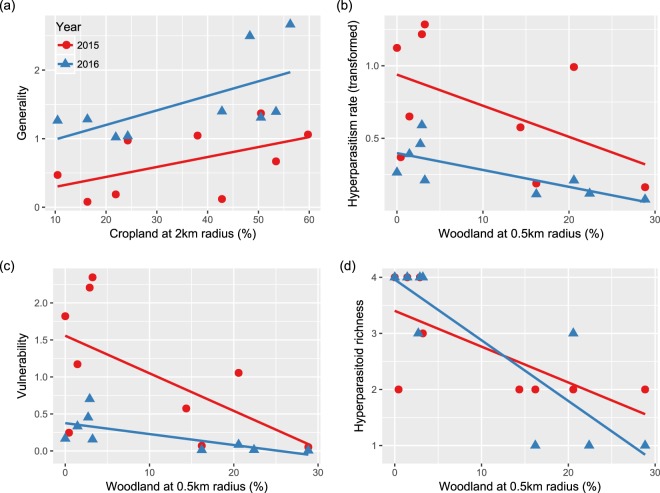
Effect of cropland and woodland percentages on food web metrics: generality (**a**), vulnerability (**c**), hyperparasitism rate (**b**) and hyperparasitoid richness (**d**). Hyperparasitism rate was arcsine-square-root transformed for analysis. Red circles and lines show results from the year 2015, while blue triangle and lines represent the year 2016. Regression lines are shown for descriptive purposes (see text for statistical results).

Generality increased with percentage cropland in both years (Fig. 2a, Table 2). Multiple comparisons of models for generality evaluated at different radii (0.5, 1, 2, 3 km) demonstrated a similar result (see supplementary materials Tables S3 and S4). Across all of the spatial scales, the AICc of the model at 2 km was lowest, indicating that this was the best fitting models for generality. Vulnerability decreased as percentage woodland increased, although this effect was only significant at 0.5 km (Fig. 2c, Table 2, see also supplementary materials Tables S3 and S4). Link density and interaction evenness were not affected by landscape composition because no landscape variables entered the best fitting models (Table 2).

**Table 2:** Summary result of model averaging (parameter estimates, S.E and 95% CI and associated p-values) for the fixed effects from the competing models predicting food web metrics, aphid mortality, active primary parasitism, hyperparasitism and richness of (hyper)parasitoids. The presented predictors are those retained in the models after model selection based on information theoretic criteria. Predictors are shown together with the radius of impact.

Response	Parameter	Estimate	S.E	Lower CI	Upper CI	z value	*p* value
**Generality**							
	(Intercept)	0.464	0.489	−0.618	1.548	0.948	0.461
	Cropland at 2 km	0.018	0.007	0.005	0.031	2.764	0.015*
**Vulnerability**							
	(Intercept)	0.842	0.481	−0.183	1.867	1.610	0.107
	Woodland at 0.5 km	−0.032	0.013	−0.061	−0.003	−2.176	<0.030*
**Link density**							
	(Intercept)	0.870	0.174	0.449	1.290	4.990	0.126
**Interaction evenness**							
	(Intercept)	0.551	0.148	0.198	0.904	3.726	0.111
**Aphid mortality**							
	(Intercept)	4.3913	0.7923	2.4792	6.3033	5.543	0.114
**Active primary parasitism**							
	(Intercept)	2.3612	0.5997	1.1112	3.6111	3.702	<0.001***
	Woodland at 0.5 km	0.0756	0.0322	0.0060	0.1452	2.128	0.033*
**Hyperparasitism**							
	(Intercept)	0.612	0.251	0.077	1.147	2.240	0.025*
	Woodland at 0.5 km	−0.016	0.007	−0.031	−0.002	2.188	0.029*
**Primary parasitoid richness**							
	(Intercept)	3.481	1.432	0.406	6.556	2.219	0.027*
	Vegetable at 1 km	−0.045	0.020	−0.089	−0.001	2.015	0.044*
**Hyperparasitoid richness**							
	(Intercept)	3.677	0.253	3.184	4.170	14.552	<0.001***
	Woodland at 0.5 km	−0.087	0.017	−0.121	−0.053	−4.991	<0.001***

*p < 0.05, **p < 0.01, ***p < 0.001.

Aphid mortality was not affected by landscape factors. Active primary parasitism increased with percentage woodland at 0.5 km, while hyperparasitism decreased with increasing percentage woodland at same scale (Fig. 2b, Table 2, see also supplementary materials Tables S5 and S6). Primary parasitoid richness decreased as percentage vegetable land increased at 1 km, while hyperparasitoid richness decreased as percentage woodland increased at 0.5 km (Fig. 2d, Table 2, see also supplementary materials Tables S5 and S6).

Aphid mortality was positively correlated with vulnerability (Pearson correlation *r* = 0.571, *p* = 0.013) but negatively correlated with generality (*r* = −0.556, *p* = 0.017). Active primary parasitism was negatively correlated with vulnerability (*r* = −0.701, *p* = 0.001), link density (*r* = −0.510, *p* = 0.031) and hyperparasitism (*r* = −0.751, *p* < 0.001). Hyperparasitism was positively correlated with vulnerability (*r* = 0.974, *p* < 0.001) but negatively correlated with both generality (*r* = −0.704, *p* = 0.001) and primary parasitoid richness (*r* = −0.536, *p* = 0.022).

## Discussion

Here, we aimed in part to determine how the food web structures of parasitoid-hyperparasitoid in greenhouses responded to landscape composition. We found that cropland increased generality (i.e., the mean number of primary parasitoid per hyperparasitoid species). The positive effect of cropland on primary parasitoids has two possible explanations. First, parasitoids might benefit from the increased availability of host resources. Parasitoids are quite closely linked with aphids based on host-parasitoid relation and aphid honeydew as food source^18^. Crops (e.g. soybean) usually harbor many aphids, which act as host for primary parasitoids. Most of the primary parasitoids identified here, such as *B*. *communis* and *Lysiphlebus fabarum*, are generalist; the possible hosts of these parasitoids include soybean aphid, *Aphis glycines* Matsumura^49^ and *A*. *gossypii*. Second, additional cropland areas might increase the chances of parasitoids migration from fields to greenhouses. Increased dispersal among habitat patches facilitates food web complexity, dispersal may thus play a determinant role in food web structure^50^. Therefore, the surrounding cropland increased the complexity of parasitoid-hyperparasitoid food web. The higher generality in crop-dominant landscapes may therefore actually be a result of bottom-up effect from aphid-parasitoid interactions outside the greenhouses. Such pattern of trophic cascade is very common in host-parasitoid systems^51^.

Aphid control was not enhanced by increased cropland percentages. In fact, aphid mortality owing to parasitism was not affected by landscape factors, probably because hyperparasitoid activity offset the function of primary parasitoid assemblage. As the hyperparasitoids tended toward polyphagy with little host specificity, they also benefited by the increase in primary parasitoids. This effect was also indicated by the hyperparasitism rate, which was positively related to aphid mortality. Biocontrol potential (i.e., active primary parasitism), was negatively correlated with hyperparasitism and positively correlated with woodland percentages at 0.5 km scale. This suggested that hyperparasitoid-focused landscape management might be a promising strategy for the improvement of biological control measures.

Woodland areas were negatively correlated with vulnerability (i.e., the mean number of hyperparasitoid per primary parasitoid species), hyperparasitoid richness and hyperparasitism. Parasitoid species were on average attacked by a fewer hyperparasitoid species in landscapes with higher percentages of woodland, perhaps because hyperparasitoid species richness was also lower in these types of landscapes. These results suggested that woodland habitats provided little for hyperparasitoids in terms of alternative hosts and other resources. The negative relationship between hyperparasitoid and woodland was significant at a small scale (0.5 km radius), supporting the view that species losses are usually greatest in the smallest fragments^52^. As spatial scale increased, more potentially suitable habitats could be available, allowing hyperparasitoids populations to persist. High parasitoid diversity can promote high rates of parasitism^53^. Therefore, high rate of hyperparasitism was related to complex consumer communities (high vulnerability). In addition, hyperparasitism was negatively correlated with generality, indicating that bottom-up control is stronger than top-down control when complexity of host community increases. Lack of relationships between landscape features and parasitoid-hyperparasitoid interactions evenness (or link density) might be explained by countervailing effects of generality and vulnerability. Overall hyperparasitism rates were lowest in landscapes with the greatest percentages of woodland habitats at 0.5 km. This finding raises the question of species-area relationship for hyperparasitoids^54^. It suggests that the scale of 0.5 km radius may be an effective scale for habitat management aimed at reducing the hyperparasitoid diversity and hyperparasitism. The food web metrics respond to landscape composition at different spatial scales. Thus, studies at multiple scales are required for the analysis of food web metrics.

In greenhouses, methods that introduce non-pest hosts on non-crop plants to maintain parasitoid populations have been widely adopted^55^. Thus, alternative prey or food sources can promote natural enemy population growth prior to pest outbreaks and increase biocontrol services. Our study is the first to analyze the effect of surrounding landscape on the food web of parasitoids in greenhouses. Food web structure in greenhouses located in different types of landscapes can be expected to differ. Management of the surrounding habitats may be a promising strategy to enhance biological control in greenhouses. Recently, methods involving the establishment of native plants around greenhouses have been studied for natural enemy conservation in a greenhouse-dominated landscape^56,57^. More importantly, these plants should increase the number of natural enemies in the surroundings and facilitate the migration of natural enemies to the greenhouses when necessary.

The difference in primary parasitoid richness between 2015 and 2016 was one of the factors driving the changes in food-web structure. The variation in richness between years may primarily result from differences in sampling duration. In 2015, sampling was conducted at the watermelon fruit stage, corresponding to the peak and decline in aphid population. However, samples were collected earlier in 2016, during the watermelon flower stage. More primary parasitoid species were identified in 2016 than in 2015, indicating that primary parasitoid primarily occurred early in the growing season. Hyperparasitoids mainly act later in the season, namely in the period when aphid population densities break down owing to decreasing resource quality^21^. This discrepancy between the appearance points of primary and hyperparasitoids should be considered in future studies. More intensive sampling, with the same number of sampling events each year, is required to cover the entire aphid infestation period; this type of sampling program might yield more details about parasitoid dynamics. Although our approach suffered greatly from the limited number of samples we were able to obtain, our results provide a valuable framework and basis for future studies.

A complex host-parasitoid food web structure does not necessarily lead to improved biocontrol. On the contrary, top-down control in host-parasitoid systems appears to be strong in simplified food webs with only one or a few key stone species^51,58^. Here, we focused on the third and fourth levels of trophic interactions (i.e., primary parasitoid and hyperparasitoid) to characterize the link between food web structure and pest control service. Our result showed that hyperparasitism could be higher when host community was less complex (lower generality) and consumer community was more complex (higher vulnerability). This may be related to simple interaction structures between hyperparasitoids and the dominant primary parasitoid *B*. *communis*. In the landscapes with high cropland proportion, parasitoid communities are possibly under bottom-up control, and thus exert little or no influence on pest control. In the landscapes with high proportion of woodland, active primary parasitism (biocontrol potential) tended to increase, and both hyperparasitoid richness and hyperparasitism decreased. This suggested that the top-down force of primary parasitoid was increased when hyperparasitoid was limited. Thus, landscape management programs that aim to increase primary parasitoid should consider the side effects of increasing the hyperparasitism, as these effects may dampen biocontrol potential. It may be useful to use landscape management techniques to improve biocontrol by targeting, and thus limiting the impact of, hyperparasitoid.

## Supplementary information

Supplementary materials
