# Supplementary material for: Food web structure of parasitoids in greenhouses is affected by surrounding landscape at different spatial scales
Source: Sci Rep. 2019 Jun 11;9:8442. doi: 10.1038/s41598-019-44857-1 (PMC6560093; doi:10.1038/s41598-019-44857-1)
Supplement: Supplementary file 1 — Supplementary materials [file 41598_2019_44857_MOESM1_ESM.pdf]

Food web structure of parasitoids in greenhouses is affected by surrounding landscape at different spatial scales

Zhaoke Dong[1], Xingyuan Men[2], Shuang Liu[1], Zhiyong Zhang[1*]

1 Beijing Key Laboratory of New Technology in Agricultural Application, National Demonstration Center for Experimental Plant Production Education, Beijing University of Agriculture, Beijing 102206, China

2 Institute of Plant Protection, Shandong Academy of Agricultural Science, Ji'nan 250100, China

[*] Correspondence: Zhiyong Zhang, e-mail: zzy@bua.edu.cn. Tel.: +86 010 80797302.

Table S1 Species names and abundances during 2015 and 2016.

Species code	Species	Sum of samplings	
		2015	2016
Primary parasitoids			
1	*Aphelinus varipes* (Förster)	10	125
2	*Aphidius gifuensis*	1	17
3	*Aphidius uzbekistanicus* Luzhetzki (Powell&Zhang,1983)	0	3
4	*Binodoxys communis* (Gahan,1927)	396	2546
5	*Diaeretiella rapae*（M'Intosh,1855）	0	5
6	*Lipolexis gracillis*	1	22
7	*Lysiphlebus fabarum*（Marshall,1896)	0	186
8	*Praon* sp.	0	1
Hyperparasitoids			
9	*Alloxysta chinensis* Fülöp＆Mikó,sp.nov.	41	59
10	*Asaphes* sp.	23	4
11	*Dendrocerus* sp.	22	9
12	*Pachyneuron aphidis* (Bouche,1834)	135	15
13	*Syrphophagus aphidivorus* (Mayr,1876)	147	143

Table S2 Pearson's correlation coefficient among landscape variables. Cell entries are n=18. ***P<0.001, **P<0.01, *P<0.05

		Cropland	Vegetable	Orchard	Grassland	Woodland
0.5km radius	Cropland		-0.55*	-0.42	-0.24	-0.48*
	Vegetable			-0.36	-0.33	0.29
	Orchard				0.80***	-0.10
	Grassland					-0.31
	Woodland					
1km radius	Cropland		-0.48*	-0.30	0.16	-0.31
	Vegetable			-0.51*	0.002	0.27
	Orchard				0.14	-0.37
	Grassland					0.01
	Woodland					
2km radius	Cropland		-0.27	-0.31	0.56*	-0.41
	Vegetable			-0.50*	-0.26	0.45
	Orchard				-0.28	-0.65**
	Grassland					-0.33
	Woodland					
3km radius	Cropland		-0.01	-0.37	0.54*	-0.24
	Vegetable			-0.58*	-0.42	0.41
	Orchard				-0.10	-0.69**
	Grassland					-0.19
	Woodland					

Table S3 List of candidate models used to evaluate the importance of landscape variables for food web metrics (generality, vulnerability, link density and interaction evenness). Only the competing models (△AICc list < 2) are shown. Each scale was analyzed separately.

Response variable	Scale (km)	Model	K	LL	AICc	△AICc	weight	Adj.r^2
Generality	0.5	(Intercept only)	4	-16.46	44.15	0.00	0.42	0.00
		Cropland	5	-19.19	44.42	0.26	0.37	0.22
		Grassland	5	-17.46	45.51	1.36	0.21	0.16
	1	Cropland	5	-17.87	42.00	0.00	1.00	0.34
	2	Cropland	5	-17.30	41.39	0.00	1.00	0.37
	3	Cropland	5	-17.50	42.12	0.00	1.00	0.34
Vulnerability	0.5	Woodland	5	-20.24	48.59	0.00	0.61	0.27
		(Intercept only)	4	-19.03	49.52	0.93	0.39	0.00
	1	(Intercept only)	4	-19.03	49.52	0.00	0.66	0.00
		Cropland	5	-21.67	50.82	1.29	0.34	0.15
	2	(Intercept only)	4	-19.03	49.52	0.00	0.64	0.00
		Cropland	5	-21.34	50.70	1.17	0.36	0.16
	3	(Intercept only)	4	-19.03	49.52	0.00	0.49	0.00
		Woodland	5	-20.17	50.49	0.97	0.30	0.17
		Grassland	5	-19.10	51.31	1.78	0.20	0.13
Link density	0.5	(Intercept only)	4	-1.77	12.93	0.00	1.00	0.00
	1	(Intercept only)	4	-1.77	12.93	0.00	1.00	0.00
	2	(Intercept only)	4	-1.77	12.93	0.00	1.00	0.00
	3	(Intercept only)	4	-1.77	12.93	0.00	1.00	0.00
Interaction evenness	0.5	(Intercept only)	4	2.54	3.89	0.00	1.00	0.00
	1	(Intercept only)	4	2.54	3.89	0.00	1.00	0.00
	2	(Intercept only)	4	2.54	3.89	0.00	1.00	0.00
	3	(Intercept only)	4	2.54	3.89	0.00	1.00	0.00

AICc -Akaike's information criterion second order.

K – number of parameters

△AICc - The difference between AICc values of the best ranked model and model i.

weight - Akaike weight of the model i

LL - log-likelihood of the model

Adj.r^2 - a coefficient of determination of model based on likelihood-ratio test

Table S4 Summary result of model averaging: Parameter estimates (β), S.E and 95% CI and associated p-values for the fixed effects after model averaging from the competing models predicting food web metrics. Significant codes: ***p < 0.001, **p < 0.01, *p < 0.05.

Response variable	Scale (km)	Parameter	Estimate	S.E	Lower CI	Upper CI	z value	Pr(>|z|)
Generality	0.5	(Intercept)	1.0470	0.4729	0.0376	2.0563	2.033	0.042*
		Cropland	0.0084	0.0043	-0.0009	0.0178	1.771	0.077
		Grassland	-0.0790	0.0429	-0.1718	0.0137	1.670	0.095
	1	(Intercept)	0.7074	0.4676	-0.3788	1.7928	1.513	0.336
		Cropland	0.0126	0.0048	0.0029	0.0224	2.624	0.019*
	2	(Intercept)	0.4638	0.4893	-0.6183	1.5478	0.948	0.4611
		Cropland	0.0179	0.0065	0.0049	0.0310	2.764	0.0145*
	3	(Intercept)	0.3338	0.5109	-0.7319	1.4097	0.653	0.5726
		Cropland	0.0203	0.0080	0.0045	0.0367	2.547	0.0223*
Vulnerability	0.5	(Intercept)	0.8419	0.4805	-0.1827	1.8666	1.610	0.107
		Woodland	-0.0322	0.0134	-0.0611	-0.0032	2.176	0.030*
	1	(Intercept)	0.7707	0.5748	-0.4555	1.9969	1.232	0.218
		Cropland	-0.0124	0.0065	-0.0265	0.0017	1.720	0.085
	2	Intercept	0.8494	0.6256	-0.4733	2.1721	1.259	0.208
		Cropland	-0.0059	0.0088	-0.0356	0.0023	1.719	0.086
	3	(Intercept)	0.8783	0.5789	-0.3480	2.1046	1.404	0.160
		Woodland	-0.0650	0.0339	-0.1384	0.0083	1.739	0.082
		Grassland	-0.1871	0.1224	-0.4514	0.0772	1.387	0.165
Link density	0.5	(Intercept)	0.8698	0.1743	0.4492	1.2904	4.99	0.126
	1	(Intercept)	0.8698	0.1743	0.4492	1.2904	4.99	0.126
	2	(Intercept)	0.8698	0.1743	0.4492	1.2904	4.99	0.126
	3	(Intercept)	0.8698	0.1743	0.4492	1.2904	4.99	0.126
Interaction evenness	0.5	(Intercept)	0.5507	0.1478	0.1977	0.9038	3.726	0.111
	1	(Intercept)	0.5507	0.1478	0.1977	0.9038	3.726	0.111
	2	(Intercept)	0.5507	0.1478	0.1977	0.9038	3.726	0.111
	3	(Intercept)	0.5507	0.1478	0.1977	0.9038	3.726	0.111

Table S5 List of candidate models used to evaluate the importance of landscape variables for active primary parasitism, aphid mortality rate, hyperparasitism, richness of primary parasitoids and richness of hyperparasitoids. Only the competing models (△AICc list < 2) are shown. Each scale was analyzed separately.

Response variable	Scale (km)	Model	K	LL	AICc	△AICc	weight	Adj.r^2
Active primary parasitism	0.5	Woodland	5	-33.32	76.34	0.00	0.67	0.26
		(intercept only)	4	-33.30	77.72	1.38	0.33	0.00
	1	(intercept only)	4	-33.30	77.72	0.00	0.71	0.00
		Vegetable	5	-34.86	79.52	1.80	0.29	0.11
	2	(intercept only)	4	-33.30	77.72	0.00	0.69	0.00
		Vegetable	5	-34.13	79.32	1.59	0.31	0.12
	3	(intercept only)	4	-33.30	77.72	0.00	0.71	0.00
		Vegetable	5	-33.87	79.49	1.77	0.29	0.12
Aphid mortality	0.5	(intercept only)	4	-35.92	84.27	0.00	1	0.00
	1	(intercept only)	4	-35.92	84.27	0.00	1	0.00
	2	(intercept only)	4	-35.92	84.27	0.00	1	0.00
	3	(intercept only)	4	-35.92	84.27	0.00	0.71	0.00
		Grassland	5	-34.95	86.06	1.81	0.29	0.11
Hyperparasitism	0.5	Woodland	5	-9.46	24.46	0.00	0.66	0.45
		(intercept only)	4	-7.83	25.78	1.32	0.34	0.00
	1	(intercept only)	4	-7.83	25.78	0.00	0.72	0.00
		Cropland	5	-11.45	27.71	1.94	0.28	0.19
	2	(intercept only)	4	-7.83	25.78	0.00	0.65	0.00
		Cropland	5	-10.83	27.00	1.22	0.35	0.25
	3	(intercept only)	4	-7.83	25.78	0.00	0.55	0.00
		Grassland	5	-8.55	27.56	1.79	0.23	0.20
		Woodland	5	-9.97	27.57	1.80	0.22	0.20
Richness of primary parasitoids	0.5	Vegetable	5	-30.31	71.36	0.00	0.53	0.21
		(intercept only)	4	-29.04	71.62	0.26	0.47	0.00
	1	Vegetable	5	-29.76	70.95	0.00	0.58	0.23
		(intercept only)	4	-29.04	71.62	0.67	0.42	0.00
	2	(intercept only)	4	-29.04	71.62	0.00	0.67	0.00
		Vegetable	5	-30.05	73.02	1.40	0.33	0.14
	3	(intercept only)	4	-29.04	71.62	0.00	0.68	0.00
		Grassland	5	-28.43	73.11	1.50	0.32	0.13
Richness of hyperparasitoids	0.5	Woodland	5	-23.35	53.68	0.00	1	0.64
	1	Woodland	5	-28.82	66.86	0.00	0.50	0.21

	(Intercept only)	4	-27.81	66.88	0.02	0.50	0.00
2	(Intercept only)	4	-27.81	66.88	0.00	1	0.00
3	(Intercept only)	4	-27.81	66.88	0.00	0.52	0.00
	Woodland	5	-28.39	67.02	0.14	0.48	0.20

AICc -Akaike's information criterion second order.

K – number of parameters

△AICc - The difference between AICc values of the best ranked model and model i.

weight - Akaike weight of the model i

LL - log-likelihood of the model

Adj,r2 - a coefficient of determination of model based on likelihood-ratio test

Table S6 Summary result of model averaging: Parameter estimates (β), S.E and 95% CI and associated p-values for the fixed effects after model averaging from the competing models predicting active primary parasitism, aphid mortality, hyperparasitism, richness of primary parasitoids and richness of hyperparasitoids. Significant codes: ***p < 0.001, **p < 0.01, *p < 0.05.

Response variable	Scale (km)	Parameter	Estimate	S.E	Lower CI	Upper CI	z value	Pr(>|z|)
Active primary parasitism	0.5	(Intercept)	2.3612	0.5997	1.1112	3.6111	3.702	<0.001***
		Woodland	0.0756	0.0322	0.0060	0.1452	2.128	0.033*
	1	(Intercept)	2.7296	0.5163	1.6394	3.8198	4.907	<0.001***
		Vegetable	0.0439	0.0309	-0.0228	0.1105	1.289	0.197
	2	(Intercept)	2.6311	0.6323	1.3111	3.9511	3.907	<0.001***
		Vegetable	0.0884	0.0602	-0.0417	0.2185	1.331	0.183
	3	(Intercept)	2.6088	0.6909	1.1700	4.0475	3.554	<0.001***
		Vegetable	0.1192	0.0847	-0.0064	0.3021	1.278	0.2013
Aphid mortality	0.5	(Intercept)	4.3913	0.7923	2.4792	6.3033	5.543	0.114
	1	(Intercept)	4.3913	0.7923	2.4792	6.3033	5.543	0.114
	2	(Intercept)	4.3913	0.7923	2.4792	6.3033	5.543	0.114
	3	(Intercept)	4.6629	0.9791	2.5908	6.7349	4.411	<0.001***
		Grassland	-0.5304	0.3518	-1.2904	0.2296	1.368	0.171
Hyperparasitism	0.5	(Intercept)	0.6119	0.2508	0.0765	1.1474	2.240	0.025*
		Woodland	-0.0164	0.0068	-0.0311	-0.0017	2.188	0.029*
	1	(Intercept)	0.5499	0.2825	-0.0533	1.1532	1.787	0.074
		Cropland	-0.0058	0.0035	-0.0133	0.0017	1.519	0.129
	2	(Intercept)	0.6085	0.3197	-0.0671	1.2840	1.765	0.078
		Cropland	-0.0087	0.0046	-0.0185	0.0012	1.723	0.085
	3	(Intercept)	0.5931	0.2886	-0.0212	1.2073	1.892	0.058
		Woodland	-0.0977	0.0637	-0.2354	0.0340	1.391	0.164
		Grassland	-0.0274	0.0179	-0.0661	0.0113	1.386	0.166
Richness of primary parasitoids	0.5	(Intercept)	3.4149	1.4527	0.2933	6.5364	2.144	0.032*
		Vegetable	-0.0295	0.0139	-0.0595	0.0004	1.935	0.053
	1	(Intercept)	3.4813	1.4318	0.4063	6.5564	2.219	0.027*
		Vegetable	-0.0449	0.0202	-0.0886	-0.0012	2.015	0.044*
	2	(Intercept)	3.3702	1.4421	0.2799	6.4606	2.137	0.033*
		Vegetable	-0.0671	0.0422	-0.1582	0.0240	1.443	0.149
	3	(Intercept)	2.9683	1.4509	-0.1412	6.0778	1.871	0.061
		Grassland	0.3469	0.2205	-0.1295	0.8232	1.427	0.154
Richness of hyperparasitoids	0.5	(Intercept)	3.6771	0.2527	3.1841	4.1701	14.552	<0.001***
		Woodland	-0.0869	0.0174	-0.1208	-0.0529	-4.991	<0.001***

1	(Intercept)	3.1027	0.5408	1.9747	4.2307	5.391	<0.001***
	Woodland	-0.0702	0.0366	-0.1493	0.0089	1.740	0.082
2	(Intercept)	2.7778	0.4444	1.7051	3.8504	6.250	0.101
3	(Intercept)	3.2587	0.7206	1.7716	4.7458	4.295	<0.001***
	Woodland	-0.1137	0.0608	-0.2449	0.0176	1.698	0.090
